# Studying the silent side of birdsong

**DOI:** 10.1186/s12915-014-0062-8

**Published:** 2014-08-06

**Authors:** Gonçalo C Cardoso

**Affiliations:** CIBIO - Centro de Investigação em Biodiversidade e Recursos Genéticos, Universidade do Porto, Campus Agrário de Vairão, Vairão, 4485-661 Portugal

## Abstract

The quality of complex communication signals, such as birdsong, is difficult to assess and compare across individuals or species. A new study on skylark song avoids the problem of signal complexity by assessing motor performance during the silent gaps of songs. This provides a metric of song quality applicable to species with very diverse songs, which facilitates novel types of analyses and comparisons in avian bioacoustics.

See research article: http://www.biomedcentral.com/1741-7007/12/58.

## Commentary

Explaining the diversity of animal communication signals is a longstanding goal for ethologists and evolutionary biologists. Sexual signals, in particular, may involve extravagant behavior or ornamentation that often differ between closely related species, making them important and challenging models for the study of communication and signal evolution. A new study on the singing behavior of skylarks - an iconic bird of the Paleartic due to its long, in-flight songs - uses a cleverly simple trick that promises to help bioacoustic research on sexual signaling in a wide variety of avian species: rather than focusing on voiced sounds, it infers vocal tract adjustments during the silent gaps between syllables [1]. Voiced sounds are often complex and idiosyncratic to each species, such that methods to study them may not apply well across species, while vocal tract modulation during silent intervals may afford metrics of vocal performance applicable to species with diverse songs.

Sexual signals such as birdsong are used for mate attraction or stimulation, and in intra-sexual competition, and in these contexts the interests of senders and receivers differ. It would, for example, be beneficial for courting males to advertise higher quality than true, or for competing individuals to boast higher competitive ability or aggressive motivation than true. Responding to such deceptive signaling would be detrimental for receivers, who are selected to disregard unreliable signals and instead attend to signals that are more informative. Functional sexual signaling thus depends on mechanisms that guarantee some degree of signal reliability, and studying those mechanisms is at the core of research in animal communication [2].

For behavioral signals, a hypothesis that has received growing attention is that signal performance reveals the neuro-muscular ability of individuals, which in turn indicates individual quality because good neuro-muscular skills imply good genes and a favorable developmental history [3]. We can try to assess signal performance using simple, one-dimensional metrics (for example, how fast is a movement, how loud is a sound), but sexual signals are often complex and differ in multiple dimensions that cannot be maximized simultaneously. Therefore, taking into account compromises and trade-offs between different signal properties should allow a more realistic assessment of signal performance. Knowledge of the trade-offs and limitations of communication signals, and whether animals push their signaling behavior close to those limits, can also shed light on the evolution of the diverse and complex communication signals of animals.

The idea of using trade-offs between signal properties to derive metrics of performance was pioneered by Jeffrey Podos, who noted a decreasing upper limit for how rapidly songbirds repeat trilled syllables as their frequency bandwidth augments [4], likely due to the time necessary to adjust vocal tract resonance during modulation of sound frequency [5]. Podos proposed that this upper limit be used as a reference to evaluate motor performance in birdsong [6]. The distance from a signal to such an upper limit (calculated for a species or a group of species) is termed ‘vocal deviation’, and has been the most used metric in studies of song performance. This metric is best applied to trilled song (repetitions of the same syllable), because consecutive syllables start at the same sound frequency, such that from the onset of a syllable to the onset of the next there must be at least one complete cycle of frequency modulation (that is, modulating away from, and then back to, the initial frequency); by contrast, when singing sequences of different syllables these cycles may or may not be complete.

The task of devising comprehensive and widely applicable metrics of song performance is daunting, because birdsong modulates sound not only with respect to frequency, but also in dimensions such as amplitude, tonal quality, and others, which are involved in a web of trade-offs. For example, various aspects of syllable complexity correlate with syllable length (thus compromising repetition rate), and sound amplitude also trades off against aspects of syllable complexity [7,8]. Ideally, a comprehensive metric of song performance would account for such trade-offs as well, so that performance could be compared across syllables or songs with different traits. A further complication is that trade-offs in song will differ across species. Species with song traits closer to their physiological limits are predicted to experience trade-offs that species with less demanding song do not experience [9], as has been shown in some cases [8]. As a consequence, more comprehensive and sophisticated metrics of performance will tend to be species-specific and less widely applicable.

**Figure 1:**
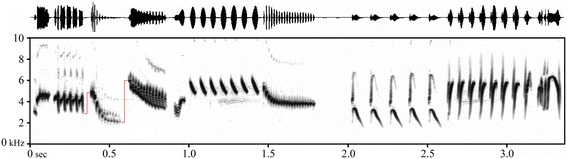
**A short section of skylark song (waveform in top panel and spectrogram in bottom panel) showing some acoustic traits that are difficult to account for in metrics of song performance: for example, fast amplitude modulations (syllable at second 1.5), prominent harmonics (at second 2), multiple frequency inflections within the syllables (at second 3.3), or differences in amplitude across syllables (top panel).** A new metric of performance avoids these difficulties by instead assessing the speed of frequency changes during the silent gaps between syllables (red lines).

In their study on skylark song, Nicole Geberzahn and Thierry Aubin take an ingenious approach: rather than tackling the problem of song complexity with increasingly sophisticated metrics of performance, they avoided the problem of complexity by looking at frequency changes during the silent gaps between syllables [1]. Their new metric, ‘vocal gap deviation’, has a similar rationale to the metric ‘vocal deviation’, only applied to the gaps between syllables; while ‘vocal deviation’ assesses motor performance as the distance to the maximum syllable repetition rate for a certain frequency bandwidth, ‘vocal gap deviation’ assesses performance as the distance to the minimum gap duration for a certain change in frequency between adjacent syllables. The gambit here was to forgo evaluating performance in the voiced portions of song, which is difficult due to various aspects of syllable complexity, and instead sample the gaps between syllables with the expectation that this gives a representative and more straightforward assessment of motor performance. Skylark song illustrates this gambit well: Figure 1 shows various properties of voiced sounds that are difficult to quantify or to incorporate in metrics of song performance (such as multiple frequency inflections within syllables, fast amplitude modulation forming buzzes or rattles, or different amplitude of syllables), but which are perceptible by bird receivers and likely used by them to evaluate song quality. During the gaps between syllables, on the contrary, there is little information available to receivers besides the duration of the silence (the time available for adjusting the vocal tract) and the change in frequency between the end of a syllable and the beginning of the next (a proxy for the extent of that adjustment). Vocal tract modulation during these gaps should be as simple as possible, because it is inaudible and complex modulation would thus serve no purpose for communication. This makes the assessment of motor performance more tractable for researchers.

Since motor performance during silent gaps is largely independent of idiosyncrasies in the sounds that flank them, ‘vocal gap deviation’ should be readily comparable across songs with different acoustic properties, or even across different species. This facilitates analyses of data where individuals sing different songs, rather than just different renditions of the same song, and enlarges the set of species among which we can compare song performance in evolutionary studies. Granting this, probably no single metric of signal performance will ever be universally applicable. In the case of ‘vocal gap deviation’, it should not be informative for species that sing widely spaced syllables whose gaps are far from a motor constraint, or when inhalations occur during singing that constrain the duration of some gaps [10] (for example, possibly the gap at second 2 in Figure 1; this issue might be circumvented by sampling gaps shorter than the minimum duration necessary for an inhalation).

As for any other metric, the value of this new method depends on whether it provides novel insights into animal communication. Geberzahn and Aubin [1] showed higher performance when skylarks sing aggressively, which in some analyses was more noticeable for ‘vocal gap deviation’ than for the simpler, one-dimensional traits that this metric is based on. That result is encouraging, and suggests that ‘vocal gap deviation’ is biologically meaningful. Together with the potential for ‘vocal gap deviation’ to be applied to, and compared among, species with diverse songs, I expect that the new method will foster innovative research in avian bioacoustics.
